# Periimplantitis: conceptos actuales sobre su etiología, características clínicas e imagenológicas. Una revisión

**DOI:** 10.21142/2523-2754-1004-2022-134

**Published:** 2023-12-26

**Authors:** Juan Carlos Martínez Gómez, Adalsa Hernández-Andara, Maira Quevedo-Piña, Ana Isabel Ortega-Pertuz, Mee Lyn Chong

**Affiliations:** 1 Facultad de Odontología, Universidad Central de Venezuela. Caracas, Venezuela. juancmartinezgomez@gmail.com Universidad Central de Venezuela Facultad de Odontología Universidad Central de Venezuela Caracas Venezuela juancmartinezgomez@gmail.com; 2 Unidad de Diagnóstico por Imagen, Clínica Félix Boada Caracas Venezuela. adalsa1@yahoo.com Unidad de Diagnóstico por Imagen Clínica Félix Boada Caracas Venezuela adalsa1@yahoo.com; 3 Facultad de Odontología, Universidad de Carabobo., Valencia Venezuela. mairaquevedo@gmail.com, meelyn_chong25@hotmail.com Universidad de Carabobo Facultad de Odontología Universidad de Carabobo. Valencia Venezuela mairaquevedo@gmail.com meelyn_chong25@hotmail.com; 4 Instituto de Investigaciones, Facultad de Odontología, Universidad del Zulia. Maracaibo, Venezuela. anitaortegav@gmail.com Universidad del Zulia Instituto de Investigaciones Facultad de Odontología Universidad del Zulia Maracaibo Venezuela anitaortegav@gmail.com

**Keywords:** implante dentario, periimplantitis, enfermedad periodontal, dental implant, peri-implantitis, periodontal disease

## Abstract

La periimplantitis (PI) es la reacción inflamatoria de la mucosa periimplantaria, acompañada de la pérdida progresiva del hueso de soporte alrededor del implante, lo que puede comprometer su estabilidad, función y estética. Es diagnosticada mediante la medición radiográfica del nivel óseo alveolar, con o sin síntomas clínicos de inflamación, y una profundidad al sondaje mayor a 4 mm. El objetivo de este trabajo fue revisar la evidencia científica sobre la prevalencia, etiología, factores predisponentes, comportamiento clínico y las características imagenológicas de la PI. Se realizó una búsqueda electrónica en Google Scholar, PubMed y SciELO, considerando el periodo comprendido entre 2010-2022, y se seleccionó un total de 40 artículos. Se concluye que el agente etiológico principal de la PI es la biopelícula; sin embargo, su aparición y severidad puede estar asociada con la presencia de factores predisponentes, como diabetes mellitus, tabaquismo, enfermedad periodontal preexistente y ausencia de mucosa queratinizada, entre otros. Clínicamente, la PI está relacionada con el sangrado al sondaje, recesión gingival y supuración. La evaluación imagenológica del defecto periimplantar es realizada rutinariamente con radiografías periapicales; el uso reciente de tomografía computarizada de haz cónico ha permitido la valoración tridimensional del defecto, aunque se han realizado consideraciones sobre la dosis de radiación para el paciente y los artefactos de imagen que pueden limitar su uso de forma extensiva.

## Abstract (en)

Peri-implantitis (PI) is the inflammatory reaction of the peri-implant mucosa, accompanied by the progressive loss of supporting bone around the implant, which can compromise its stability, function and aesthetics. It is diagnosed by radiographic measurement of the alveolar bone level, with or without clinical symptoms of inflammation and a probing depth greater than 4 mm. The objective of this work was to review the scientific evidence on the prevalence, etiology, predisposing factors, clinical behavior and imaging characteristics of PI. An electronic search was carried out in Google Scholar, PubMed and Scielo, considering the period between 2010-2022, and a total of 40 articles were selected. It is concluded that the main etiological agent of PI is the biofilm, however, its appearance and severity may be associated with the presence of predisposing factors such as Diabetes Mellitus, smoking, pre-existing periodontal disease and absence of keratinized mucosa, among others. Clinically, PI is related to bleeding on probing, gingival recession, and suppuration. Imaging evaluation of the peri-implant defect is routinely performed with periapical radiographs; Recent use of cone-beam computed tomography has allowed three-dimensional assessment of the defect, although considerations of radiation dose to the patient and image artifacts have been made that may limit its extensive use.

## INTRODUCCIÓN

La rehabilitación bucal de espacios parcial o completamente edéntulos con implantes dentarios es una terapéutica establecida y altamente exitosa, con una sobrevivencia del 89-90% sobre los 10 años de función. Sin embargo, los implantes son susceptibles de enfermedad inflamatoria periimplantaria que puede comprometer su estabilidad, función y estética ^1^^,^^2^. Durante el taller mundial de la Asociación Americana de Periodoncia y la Federación Europea de Periodoncia, celebrado en 2017, fue incluida una nueva clasificación para las condiciones y enfermedades periimplantarias, con la finalidad de alcanzar un consenso que pudiera ser aceptado mundialmente. Estas fueron clasificadas en mucositis periimplantaria y periimplantitis (PI) ^3^. 

La mucositis periimplantaria se define como la inflamación reversible de los tejidos blandos alrededor de un implante, asociada con la presencia de biopelícula. La PI es la reacción inflamatoria de la mucosa periimplantaria con la pérdida progresiva del hueso de soporte alrededor del implante, que puede ser diagnosticada mediante la medición radiográfica del nivel óseo alveolar, con o sin síntomas clínicos de inflamación, y profundidad de sondaje mayor a 4 mm ^3^. Otros signos comunes asociados son purulencia, la disminución de la oseointegración del implante y el aumento de la formación de bolsas ^1^.

Las enfermedades periimplantarias han demostrado una tasa de prevalencia media ponderada del 43 % en Europa y del 22 % en América del Sur y del Norte ^4^. Aunque el principal agente etiológico de la PI es la biopelícula ^5^, diversos factores influyen en el inicio y progresión de la enfermedad, entre los cuales se pueden mencionar una higiene bucal deficiente, la presencia de cemento de restauración en el implante, antecedentes de enfermedad periodontal, o enfermedades sistémicas como la diabetes mellitus (DM), la esclerodermia, la displasia ectodérmica, el liquen plano, la osteoporosis, la artritis reumatoide y el síndrome de Sjögren ^6^^-^^8^. Aunque la relación entre el tabaquismo y la PI es controvertida, se ha reportado que existe una diferencia estadísticamente significativa en la incidencia de PI en el paciente fumador. Otros indicadores de riesgo identificados incluyen el diseño, el ángulo de emergencia y el tiempo de función del implante. También se han asociado con la PI los hábitos parafuncionales del paciente, la sobrecarga oclusal, los antecedentes de falla del implante o la posición incorrecta del mismo ^9^.

La evaluación longitudinal de la pérdida ósea periimplantar se ha realizado rutinariamente mediante radiografías periapicales empleando la técnica del paralelismo para estandarizar la obtención de la imagen, y su limitante principal es el registro bidimensional de una estructura tridimensional ^1^^,^^2^^,^^10^. Las radiografías panorámicas han sido utilizadas en menor grado, posiblemente debido a su baja resolución de imagen y magnificación, además de las limitaciones comunes a las técnicas en 2D ^2^^,^^10^. El uso de tomografía de haz cónico (TCHC) para evaluar la morfología y profundidad del defecto óseo en la PI es de reciente data ^2^^,^^11^^,^^12^; sin embargo, su precisión diagnóstica puede estar limitada por artefactos de imagen generados por la presencia del implante ^13^^,^^14^, ruido y bajo contraste para los tejidos blandos ^15^. 

Desafortunadamente, a pesar de recibir atención de apoyo regular, ciertos pacientes pueden requerir un nuevo tratamiento, terapias complementarias o la extracción del implante debido a la progresión o recurrencia de la enfermedad ^16^. La presente revisión tiene como objetivo conocer los diferentes aspectos a considerar en la PI en cuanto a su definición, prevalencia, etiología, factores predisponentes, así como su comportamiento clínico y las características imagenológicas, lo que le permitirá al clínico evaluar los factores que pueden predisponer a la inflamación del tejido periimplantario y decidir, entre un conjunto de posibilidades, el tratamiento requerido para evitar la enfermedad y sus consecuencias. 

## MATERIALES Y MÉTODOS

Se realizó una búsqueda electrónica en Google Scholar, PubMed y SciELO, utilizando las palabras clave: peri-implantitis/riskfactors/prevalence/etiology/diagnostic imaging/cone beam CT. La selección de los artículos se efectuó considerando los siguientes criterios de inclusión: documentos en extenso publicados en idioma inglés, español o portugués, que correspondieran a investigaciones realizadas en humanos o modelos animales, de tipo observacional, aleatorizadas/controladas, revisiones sistemáticas/metanálisis, consensos e informes. Los criterios de exclusión fueron los siguientes: reportes de casos clínicos, revisiones narrativas u opiniones de expertos. Los datos de las publicaciones relevantes fueron extraídos y se organizaron en tablas. Una vez leídos, se procedió a filtrar los artículos de acuerdo con su pertinencia directa con el tema a abordar y se descartaron las publicaciones dobles. Finalmente, la información se resumió según las consideraciones seleccionadas como objeto de análisis para el manuscrito. Se abarcó el período comprendido desde 2010 hasta 2022, inclusive. Los detalles de la estrategia de búsqueda se resumen en las Tablas 1 y 2.

**Tabla 1:** Estrategia de búsqueda

Historial de búsqueda	Investigaciones encontradas
Revisión preliminar utilizando palabras clave * y términos libres	352
Eliminación de estudios repetidos	48
Lectura de *abstract* y resumen	352
Selección de artículos publicados en inglés y español	73
Lectura de artículos completos	73
Selección de artículos relevantes al estudio	40

* Identificadas en Medical Subject Headings (MeSH) y Descriptores en Ciencias de la Salud (DeCS).

**Tabla 2:** Distribución de los artículos seleccionados según su tipo

Tipo de estudio	Número de investigaciones encontradas
Consensos e informes	6
Estudios observacionales	20
Casos y control	2
Revisiones sistemáticas	12
Total	40

## RESULTADOS Y DISCUSIÓN

### Definición

La PI se define como una condición patológica asociada a la biopelícula, que ocurre en el tejido alrededor de los implantes dentales, caracterizada por inflamación en la mucosa periimplantaria y la subsiguiente pérdida progresiva del hueso de soporte ^6^. Se ha observado que la mucositis periimplantaria precede a la PI, la cual se asocia con un control deficiente de la biopelícula y pacientes con antecedentes de periodontitis grave. El inicio de la PI puede ocurrir temprana o medianamente después de la colocación del implante, y en ausencia de tratamiento, parece progresar con un patrón acelerado y no lineal ^17^, que puede deberse a la interacción entre los microorganismos presentes en el sitio del implante, el mecanismo de defensa del huésped y la ausencia de ligamento periodontal ^8^. Representa una infección mixta heterogénea que incluye anaerobios gramnegativos, bacilos grampositivos anaerobios asacarolíticos y, en raras ocasiones, microorganismos oportunistas como bastoncillos entéricos y *Staphylococcus aureus*^18^.

El criterio mayormente usado para describir la patología periimplantaria es el sangrado al sondaje (SAS), el cual es un indicador común de una lesión inflamatoria en los tejidos circundantes de los dientes naturales y se ha sugerido como una medida diagnóstica para la salud periimplantaria. El segundo parámetro diagnóstico incluido en la mayoría de las definiciones de PI es la profundidad al sondaje (PAS) periimplantario, como una medida del registro de la pérdida de inserción y hueso de soporte. El tercer parámetro diagnóstico de la PI es la pérdida de hueso periimplantario evaluada a través de radiografía ^19^. En este sentido, Francetti *et al*. ^20^ utilizaron como evidencia para el diagnóstico de PI la presencia de sangrado/supuración y el proceso de reabsorción ósea concomitante de 2 mm o más observado radiográficamente, sin considerar la profundidad del sondaje periimplantario. 

### Etiología

Al igual que las enfermedades periodontales, el principal factor causante de las enfermedades periimplantarias es la biopelícula ^8^. La salud periodontal está influenciada por varios factores como la higiene bucal, factores genéticos y epigenéticos, sistémicos y nutrición ^8^. Las lesiones periimplantares y periodontitis albergan una mayor proporción de bacterias anaerobias gramnegativas en comparación con los sitios sanos. Sin embargo, la PI tiene una mayor diversidad microbiana que la periodontitis, se observa el predominio de células inflamatorias, linfocitos B y células plasmáticas, con mayor proporción de leucocitos polimorfonucleares en compartimentos perivasculares en las áreas más centrales del infiltrado de células inflamatorias; de igual manera, presenta macrófagos y con frecuencia se observa que carece de una capa protectora de tejido sobre el hueso, la cual suele estar presente en la periodontitis ^8^^,^^9^. 

Histológicamente, las lesiones de PI muestran mayor tamaño y cantidad de vasos sanguíneos e infiltrado en el tejido conectivo, en comparación con la periodontitis. La tasa de progresión de la enfermedad es más rápida en la PI, lo que genera una pérdida ósea más rápida y severa en comparación con la enfermedad periodontal. En la PI se produce una forma no lineal de destrucción ósea progresiva, que puede deberse a los diversos microorganismos en los sitios de implante, el mecanismo de defensa del huésped y la ausencia de un ligamento periodontal ^8^.

Se ha reportado que las bacterias periodontopatógenas asociadas con la periodontitis alrededor de los dientes naturales son diferentes a las presentes en la PI, *Staphylococcus* spp. entéricos y *Candida* spp. han sido identificadas en el 55% de las lesiones periimplantarias. A partir de una comparación microbiológica entre implantes sanos y sitios de PI, se encontraron 19 especies en recuentos más altos en PI, lo que demuestra que un grupo bacteriano puede estar asociado con su aparición, es así como los recuentos bacterianos de *Tannerella forsythia*, *Treponema denticola*, *Treponema socranskii*, *Porphyromonas gingivali*, *Staphylococcus aureus*, *Campylobacter rectus*, *Campylobacter gracilis* y *Prevotella intermedia* tuvieron niveles significativamente mayores en términos de razones de probabilidad para sujetos con PI en comparación con implantes sin afectaciones ^21^. Igual comportamiento para la *Cándida* spp. y otros organismos fúngicos que se encontraron con frecuencia en recuentos más altos en los sitios de PI en comparación con los sitios sanos, lo que sugiere además que estos recuentos de microflora pueden desempeñar un papel en el inicio de la enfermedad ^22^.

### Prevalencia

Sobre la prevalencia de la PI existen diversas opiniones que dependen básicamente del protocolo de la investigación. Rocay *et al*. ^8^ encontraron en su estudio que la prevalencia media de la PI basada en implantes y sujetos fue del 9,25 % y el 19,83 %, respectivamente, mientras que Rinke *et al*. ^41^ detectaron PI en el 18% de los pacientes estudiados. 

Derks y Tomasi ^4^ relatan una prevalencia de PI entre el 1% y el 47% en los sujetos tratados con prótesis implantosoportadas. Francetti *et al*. ^20^ evaluaron un total de 384 implantes colocados en 77 pacientes durante un período medio de ocho años desde la carga; después de 10 años, la tasa acumulada de implantes libres de PI fue del 86,92%. Los valores citados con referencia a la prevalencia de PI presentan un amplio rango debido a que dependen de múltiples factores, entre los que pueden citarse el tiempo de permanencia del implante en boca, las condiciones del paciente y los factores de riesgo implicados, así como las diferentes definiciones diagnósticas a criterio de los investigadores para su estudio.

### Factores de riesgo y características clínicas

En esta revisión narrativa se clasificaron los factores de riesgo de PI en sistémicos y locales, y se seleccionaron aquellos que de acuerdo con la literatura consultada tienen mayor impacto en su desarrollo. Es importante destacar que en la clasificación de las enfermedades periodontales y periimplantarias de 2017 se incorporan la DM y el tabaquismo como factores de riesgo que pueden modificar el grado de la condición periodontal ^3^. 

#### Factores de riesgo sistémicos

a. Diabetes mellitus. La hiperglucemia podría ser un factor potencialmente importante en el desarrollo de complicaciones biológicas de los implantes, como se observa en la diabetes mal controlada. Los niveles elevados de glucosa en sangre producen productos finales de glicación avanzada que van a activar la expresión de su receptor y contribuyen a la reparación deteriorada de los tejidos periodontales que se descomponen por la inflamación exacerbada y sostenida causada por la biopelícula ^24^. Es así como los procesos de cicatrización pueden verse afectados negativamente por niveles elevados de glucosa en sangre, con alteraciones en la vascularización, la remodelación ósea y el aumento de la susceptibilidad a las infecciones, lo que lleva a la conclusión de que la hiperglicemia tiene efectos adversos sobre la integración del implante ^24^^,^^25^.

La DM ha sido estudiada como un factor modificador importante en la periodontitis y para algunos autores en la PI ^12^^,^^13^. En este sentido, Al-Sowygh *et al*. ^26^, encontraron que con el aumento de la hemoglobina glicosilada A1c podía observarse un deterioro significativo en los indicadores clínicos de PI, tales como > 8% en profundidades de sondaje aumentadas, sangrado al sondaje y reabsorción ósea alrededor del implante. 

En el metanálisis realizado por Dreyer *et al*. ^19^ se identificó una asociación positiva entre la DM y la PI. Los pacientes con DM presentaban dos veces más probabilidades de tener PI en comparación con los pacientes que no padecían dicha enfermedad. De igual manera, Dioguardi *et al*. ^25^ afirman que existe una mayor tasa de fracaso en el grupo de pacientes con DM y una pérdida de hueso marginal superior a la encontrada en los individuos sin DM. 

En contraposición, la revisión sistemática de Monje *et al*. ^24^ revela que ningún estudio informó la prevalencia de enfermedades periimplantarias en pacientes con hiperglucemia, sin la presencia de otros factores de riesgo tales como tabaquismo, antecedentes recientes de periodontitis o deficiente control de la biopelícula. Por lo tanto, según la información obtenida, la correspondencia entre la DM y las enfermedades periimplantarias sigue siendo explorada y la literatura existente no es conclusiva al respecto ^26^. 

b. Tabaquismo. El tabaquismo modifica varios aspectos de la inmunidad innata y adaptativa del huésped. Los fumadores muestran un aumento en el número de granulocitos y el número total de leucocitos, el incremento de la vida de las células polimorfonucleares, la producción de peróxido de hidrógeno y de inhibidores de la proteasa, así como la expresión de integrinas. La respuesta inmunitaria humoral también se ve alterada por el tabaquismo ya que inhibe la proliferación o función de los linfocitos B y T ^9^. En cuanto a los aspectos microbiológicos, Tsigarida *et al*. ^27^ encontraron mayor concentración de microbiota patógena en el entorno periimplantario de fumadores, afectados o no por enfermedad inflamatoria, a similitud de la investigación de Pimentel *et al*. ^28^, quienes observaron una tendencia de la flora patógena en fumadores, en particular de *T. forsythia*, *Fusobacterium* spp. y *Treponema*. Fumar afectó negativamente el microbioma periimplantario, lo que provocó un estado asociado a la enfermedad, incluso en individuos clínicamente sanos. 

El mecanismo por el cual el tabaco afecta la oseointegración y la supervivencia de los implantes permanece parcialmente desconocido. Sin embargo, las fallas generalmente ocurren debido al depósito de tejido fibroso en la interfase hueso-implante. El reclutamiento de preosteoblastos, su anclaje, adhesión, propagación, proliferación y diferenciación en osteoblastos, que secretan la matriz para la calcificación en la superficie del implante durante la osteointegración, es sensible a los efectos locales y sistémicos de la nicotina y otros componentes asociados del cigarrillo. La nicotina también puede inducir obstrucción microvascular, reduce el flujo sanguíneo y el suministro de nutrientes del implante en el sitio quirúrgico, así como inhibe la proliferación de fibroblastos, eritrocitos y macrófagos, afectando la regeneración ósea. Se ha especulado que a pesar de que la nicotina se expresa mínimamente en el marco de la cirugía de implantes dentales, su influencia sobre el fracaso temprano del implante está posiblemente asociado a este efecto vasoconstrictor ^29^.

Otro punto importante a considerar es que, a pesar de que fumar es considerado un factor de riesgo para la PI, no todos los pacientes fumadores la desarrollan ni todos los pacientes con enfermedad periimplantaria son fumadores. Este hecho está claramente justificado basado en la etiología multifactorial de la PI. Entre los pacientes que sufren de periimplantitis, comparando los fumadores y no fumadores de cigarrillos; estos últimos mostraron los mejores resultados a largo plazo después de un año de seguimiento del tratamiento ^29^. 

#### Factores de riesgo locales

Entre los factores de riesgo locales considerados en la clasificación de 2017 que promueven el desarrollo de la PI se consideran la historia previa de periodontitis, el control deficiente de la biopelícula y el incumplimiento de las visitas de mantenimiento posteriores a la terapia con implantes ^3^. La ausencia de mucosa queratinizada (MQ) alrededor del implante también es considerada un factor de riesgo ^30^. Del mismo modo, se ha relacionado la PI con la presencia de cemento submucoso posrestauración, defectos en el posicionamiento de los implantes que no facilita la higiene bucal y su mantenimiento, así como la sobrecarga oclusal, la necrosis por compresión ósea, el sobrecalentamiento en el tiempo quirúrgico, los micromovimientos y la biocorrosión del material del implante ^3^^,^^5^^,^^17^.

a. Historia previa de periodontitis. La literatura existente apunta a similitudes entre las enfermedades periodontales y periimplantarias en términos de etiología, patogenia, factores de riesgo y presentación clínica. La iniciación de las dos patologías depende de la presencia de la biopelícula y se han observado pocas diferencias cualitativas entre las que se encuentran en la enfermedad periodontal y la PI ^16^. Existe una fuerte evidencia en estudios longitudinales y transversales de que los pacientes con antecedentes de periodontitis son susceptibles a la PI ^6^^,^^19^.

Marrone *et al*. ^31^ evaluaron la prevalencia de mucositis y PI en individuos con implantes con al menos cinco años de funcionamiento, en pacientes con una edad promedio de 62 años. Se registraron datos generales de salud, tabaquismo, visitas de mantenimiento e higiene bucal. Se evaluó clínicamente el índice de placa, sangrado y PAS, mientras que el estado periodontal y de los implantes se determinó con radiografías panorámicas. Los resultados evidenciaron que la prevalencia de mucositis y PI a nivel de paciente fue del 31% y el 37%, respectivamente, y a nivel de implante, del 38% y el 23%. El riesgo en los sujetos mayores de 65 años fue de un OR = 1,39 (Odd ratio) y en los pacientes con periodontitis activa fueron propensos a la periimplantitis (OR = 1,98). 

Si bien se ha planteado que existe una asociación entre la periodontitis y la PI ^19^, la investigación efectuada por Ronk *et al*. ^32^ arrojó resultados diferentes. Se estudiaron 134 pacientes con 478 implantes instalados durante un período de 10 años (2001-2010), sus resultaron indicaron que, después de un período de carga de cinco años sin ningún programa de mantenimiento regular, uno de cada cinco pacientes experimentaría PI; por ello, concluyeron que ni la periodontitis presente ni sus antecedentes de ésta fueron predictores estadísticamente significativos de PI.

b. Mucosa queratinizada alrededor del implante. El tejido blando alrededor de los implantes dentales es considerablemente diferente al de los dientes naturales, tanto desde el punto de vista anatómico como histológico. En los implantes, las fibras de colágeno se distribuyen paralelas a su superficie sin anclaje directo y esta unión débil proporciona un sello biológico de calidad inferior; es por esa razón que los tejidos blandos periimplantarios son más propensos a las enfermedades inflamatorias que los dientes naturales ^33^. 

Así como la presencia de encía alrededor de los dientes naturales es importante para mantener la salud, también puede ser favorable tener una banda de MQ alrededor de los implantes dentales ^33^. Se ha indicado que la ausencia o la reducción del ancho de la MQ puede afectar negativamente las medidas de higiene bucal realizadas por el paciente en presencia de implantes; en ese sentido, Ueno *et al*. ^30^, confirmaron en un análisis transversal en 60 pacientes que una MQ <2 mm se asoció con niveles de biopelícula significativamente más altos y un aumento de los valores de sangrado al sondaje (SAS) y la PAS en comparación con los sitios de implantes con una MQ de ≥2 mm.

En su estudio transversal, Rinke *et al*. ^23^ investigaron si los indicadores de riesgo comunes para las enfermedades periimplantarias estaban asociados con mucositis periimplantaria y PI en pacientes sometidos a terapia de implante de apoyo (TIA) al menos cinco años después de la restauración del implante. Los resultados expresaron que el tabaquismo y la ausencia de MQ eran los indicadores de riesgo más fuertes de PI en pacientes sometidos a TIA. La investigación de Gharpure *et al*. ^33^, realizada en 73 pacientes con 193 implantes (seguimiento medio de 6,9 ± 3,7 años) sobre la relación del fenotipo gingival delgado (FGD) y el ancho de la MQ (AMQ) <2 mm como indicadores de riesgo de PI y mucositis, concluyó que de FDG y el AMQ inadecuados (<2 mm) pueden ser indicadores de riesgo significativos de enfermedad periimplantaria, juntamente con dolor o malestar durante el cepillado. 

Por su parte, Lin *et al*. ^34^ evaluaron el impacto del ancho de la MQ alrededor de los implantes dentales en los resultados terapéuticos quirúrgicos al tratar la PI. Se encontró que el resultado quirúrgico estuvo influenciado por la gravedad de la pérdida ósea presente en el momento del tratamiento y no por la presencia de MQ en el momento del tratamiento, e indicaron que la banda de MQ no es indispensable para el mantenimiento del tejido periimplantario.

#### Características clínicas

El diagnóstico de la PI está basado en parámetros clínicos y hallazgos radiológicos. Clínicamente, la herramienta más importante es el sondaje periodontal, el cual es utilizado para registrar el aumento de la profundidad al sondaje, la recesión gingival, sangrado y supuración ^1^^,^^5^. El sondaje puede proporcionar información precisa sobre la dimensión del defecto óseo; sin embargo, se ha reportado una variación significativa en la profundidad detectada, relacionada con el estatus de la inflación y la fuerza aplicada por el operador ^1^. 

De acuerdo con Renvert *et al*. ^35^, para el diagnóstico de la PI, se debe evidenciar el aumento de PAS de las cavidades en comparación con las medidas obtenidas en la colocación de la estructura del implante, la pérdida ósea progresiva en relación con el nivel del hueso radiográfico y su evaluación al cabo de un año de la instalación de la reconstrucción protésica implantosoportada. En ausencia de radiografías iniciales y medidas de la profundidad al sondaje, se observará evidencia radiográfica de la disminución de nivel óseo ≥3 mm y profundidad al sondaje ≥6 mm, junto con un sangrado profuso.

La PI ha sido clasificada según su morfología, por Monje *et al*. ^36^, en Clase I: defecto infraóseo; Clase Ia: dehiscencia vestibular; Clase Ib: defecto circunferencial; Clase II: defecto supracrestal; Clase III: defecto combinado; Clase IIIa: dehiscencia vestibular + pérdida ósea horizontal; Clase IIIb: defecto de 2-3 paredes; y Clase IIIc: defecto circunferencial+ pérdida ósea horizontal. Asimismo, los autores describen una subclasificación de la severidad del defecto basada en su profundidad desde el cuello del implante considerando la proporción entre la pérdida ósea total/longitud del implante: Grado S: leve 3-4 mm/ <25% de la longitud del implante; M: moderada 4-5 mm/ ≥25-50% de la longitud del implante; A: avanzada >6 mm/ 50% de la longitud del implante. 

En sitios de implantes el SAS evidencia la existencia de inflamación y demuestra la respuesta del huésped a la biopelícula, de manera que su incidencia aumenta con la severidad (mucositis periimplantar versus PI), mientras su ausencia es un fuerte indicador de la estabilidad del tejido periimplantario ^37^. Por otra parte, una tasa marcadamente alta de falsos positivos de SAS ha sido encontrada al identificar la PI. En este sentido, Hashim *et al*. ^38^ encontraron en su metaanálisis que, en el caso de los implantes positivos para SAS, existió una probabilidad del 24,4% de ser diagnosticado con PI, mientras que en los pacientes con SAS positivo se observó un 33% de probabilidad de diagnóstico de PI. En los implantes, el SAS puede tener su origen en dos formas de sondaje: inducción traumática o patológica ^37^^,^^38^. El SAS patológico, a menudo, es inducido por inflamación y podría considerarse como un indicador de enfermedad ^64^.

La supuración (SUP) acompañada de SAS es un indicador definitivo de enfermedad activa, y es sugestivo de un alto grado de inflamación y lesiones periimplantares avanzadas ^37^. Al respecto, Monje *et al*. ^39^ estudiaron las características del paciente y los sitios con implantes entre individuos que presentaban SUP y encontraron que esta fue más frecuente en vestibular del implante (51%), y mostró una menor prevalencia en el sitio mesio-lingual (16,7%). Los DPs clase Ib estuvieron asociados con la presencia de SUP (OR = 6,59; p = 0,004), cada mm adicional de profundidad al sondaje estuvo asociado con un 63% de aumento de riesgo de SUP (OR = 1,63; p = 0,024), de igual manera, cada mm de incremento de pérdida del hueso marginal aumentó el riesgo de SUP en un 35%. 

Debido a la falta de un índice para monitorear y evaluar el estatus periimplantar que incorpora el SAS, así como los componentes de biopelícula y mucosa, Dukka *et al*. ^37^ propusieron un “índice de sangrado al sondaje periimplantar”:

• Escore 1: mucoso normal, sin biopelícula visible, sin sangrado.
• Escore 2: mucositis periimplantar leve, biopelícula mínima, eritema leve con sangrado visible mínimo.
• Escore 3: mucositis periimplantar moderada, biopelícula visible, eritema evidente o edema con una línea de sangrado o goteo.
• Escore 4: mucositis periimplantar severa o PI, biopelícula evidente, eritema severo o edema con ulceración, sangrado espontáneo o profuso con o sin supuración.

Aunque la pérdida ósea es clave para definir la PI, es difícil considerarla como la única característica de diagnóstico; la reabsorción ósea puede ser causada por varios cambios fisiológicos o inducidos (p. ej., inserción profunda del implante o distancia insuficiente implante-implante/diente). Además, no todos los defectos periimplantarios > 5 mm pueden considerarse signos claros de PI ^5^^,^^6^. 

### Evaluación imagenológica

La evaluación precisa de la morfología y el tamaño del defecto periimplantar (DP) es de vital importancia clínica debido a que tiene influencia en la supervivencia del implante y el resultado terapéutico del tratamiento quirúrgico o no quirúrgico ^38^. Los DP, frecuentemente, cursan con un componente infraóseo, pérdida del hueso vestibular y diversas configuraciones morfológicas, y su severidad, a menudo, depende de estas últimas ^39^. La pérdida ósea inicial se produce en vestibular del implante debido a la relativa falta de espesor en el área ^40^.

El diagnóstico imagenológico de lesiones periimplantares se ha basado en radiografías periapicales (RP), panorámicas y, más recientemente, TCHC ^1^^,^^2^^,^^10^^,^^38^. El estudio de la precisión y confiabilidad del método de imagen en la detección, caracterización morfológica y la medición del DF ha sido realizado in vitro en modelos animales, humanos, artificiales y pacientes ^1^^,^^3^^,^^11^^-^^15^^,^^38^^,^^40^. La influencia de los factores de adquisición en la imagen de TCHC y su impacto en la valoración de la PI también ha sido valorada ^11^^,^^13^^,^^14^. 

### Radiografías periapicales

Las RP muestran el nivel del hueso interproximal en mesial y distal del implante, lo que permite detectar defectos verticales ^10^^,^^11^; sin embargo, debido a la distorsión geométrica y la sobreposición de estructuras anatómicas, el compromiso de las tablas óseas vestibular y lingual no puede ser valorado ^11^. Estas radiografías son obtenidas mediante la técnica del paralelismo para garantizar la reproducibilidad del resultado ^40^. Adicionalmente, y con propósitos de investigación, se utilizan plantillas de registro de la oclusión del paciente que estandarizan la obtención de las imágenes en diferentes momentos; la plantilla suele ser confeccionada en silicona, cera o resina. Asimismo, con el devenir de la radiología digital, existe la posibilidad de sobreponer dos imágenes seriadas y, por técnicas de sustracción, cuantificar los cambios óseos ^34^.

Las mediciones de la profundidad y ancho del DF realizadas mediante RP han demostrado una variabilidad significativa en la reproducibilidad inter e intraobservador; asimismo, se ha reportado una subestimación de la pérdida ósea ^1^^,^^2^, lo que parece estar influenciado por el tamaño del defecto y su tipo ^10^. 

### Tomografía computarizada de haz cónico

**Figura 1:**
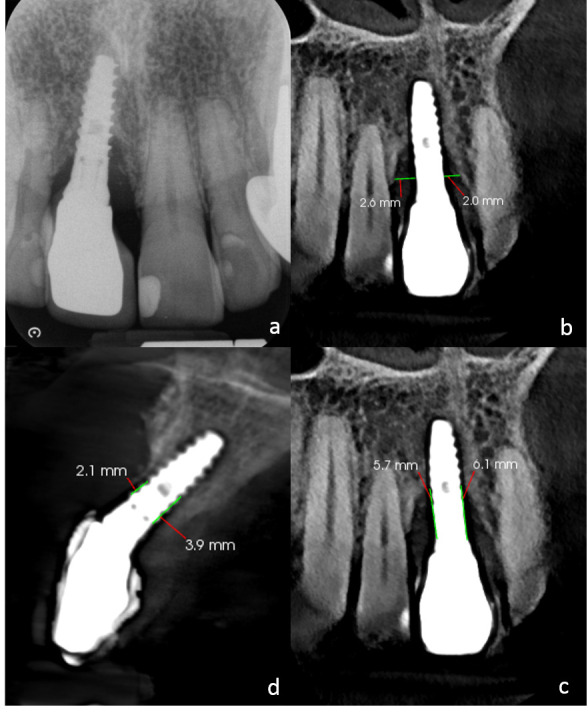
(a) Radiografía periapical digital donde se observa defecto vertical en mesial y distal del implante. Reconstrucciones multiplanares de tomografía computarizada de haz cónico con aplicación de algoritmo para la reducción de artefacto, que muestran en cortes coronales (b y c), las mediciones obtenidas de la profundidad y ancho del defecto óseo en mesial y distal del implante, en el corte sagital (d), se evidencia el compromiso de la tabla ósea vestibular (dehiscencia).

La TCHC proporciona una evaluación tridimensional de la topografía del hueso alveolar y es la modalidad de imagen estándar para la planificación de implantes y la valoración de las complicaciones postoperatorias ^13^^,^^14^ (Figuras 1 y 2), siendo la presencia de artefactos generados por el implante, como líneas hiperdensas “florecientes” que se irradian desde el mismo o bandas hipodensas adyacentes a éste, una posible limitante en la estimación del DP ^1^^,^^2^. 

**Figura 2:**
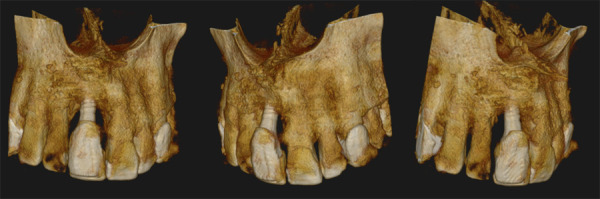
Reconstrucciones volumétricas 3D de tomografía computarizada de haz cónico donde se demuestra el defecto óseo periimplantar.

Las RP y la TCHC han sido comparadas en la valoración de DP ^12^^,^^40^. Ritter *et al*. ^12^ no encontraron diferencias estadísticamente significativas entre los valores obtenidos del nivel óseo en mesial y distal del implante; aunque ambos métodos fueron igualmente precisos, subestimaron dicho nivel. Pelelekos *et al*. ^15^ reportaron valores de sensibilidad y especificidad (sen./espec.) en la detección de dehiscencias por RP de 0,53/0,93 y 0,67/0,90 en defectos circunferenciales, la TCHC mostró valores por encima de 0,95 tanto en la sensibilidad como la especificidad en ambos tipos de defectos. 

Con el objetivo de corregir los artefactos en la TCHC, los fabricantes de equipos han desarrollado algoritmos o “filtros” de reducción. Los resultados de la aplicación de algoritmos para reducir artefactos generados por objetos metálicos (*Metal artifact reduction*-MAR) son controvertidos ^2^^,^^13^^,^^14^. En este sentido, Kamburogluk *et al*. ^13^ indicaron que no encontraron diferencias entre las imágenes de TCHC obtenidas con un tamaño de voxel de 0,200 mm al utilizar distintos tipos de MAR en la detección de defectos vestibulares periodontales o periimplantares. Por su parte, de Acevedo Vaz *et al*. ^14^ compararon dos protocolos de adquisición que utilizaban tamaños de voxel diferentes (0,200 mm y 0,300 mm) con la aplicación de MAR, y señalaron que el uso de MAR no mejoró el diagnóstico de fenestraciones o dehiscencias periimplantares, independientemente del tamaño del voxel. 

Protocolos de TCHC de “baja dosis” (TCHC-BD) han probado ser útiles en la evaluación quirúrgica y preimplantar, al ofrecer resultados aceptables en términos de calidad de imagen con la reducción de la dosis de radiación para el paciente. Leisner *et al*. ^11^ señalaron que, al comparar las mediciones de la profundidad y ancho de DF, realizadas en imágenes adquiridas por medio de TCHC de alta resolución (TCHC-AR), RP y dos protocolos de TCHC-BD, las desviaciones absolutas entre la TCHC-AR y la RP fueron de 0,32 y 0,35 mm (profundidad y ancho del defecto respectivamente), los valores obtenidos con el primer protocolo de “baja dosis” fueron de 0,18 y 0,22 mm y de 0,17 y 0,18 mm para el segundo. Por tanto, ambos protocolos de “baja dosis” fueron más precisos que la RP, al ofrecer una reducción de la dosis de radiación comparados con la TCH-AR, sin una pérdida sustancial de precisión. 

## CONCLUSIONES

La literatura disponible indica que el principal factor causante de la enfermedad periimplantar es la biopelícula; sin embargo, su aparición y severidad puede estar asociada con la presencia de factores predisponentes como la diabetes mellitus, el tabaquismo, la enfermedad periodontal preexistente y la ausencia de mucosa queratinizada alrededor del implante, entre otros. Clínicamente, la periimplantitis está relacionada con sangrado al sondaje, recesión gingival y supuración. La evaluación imagenológica del defecto periimplantar es realizada rutinariamente con radiografías periapicales; el uso reciente de tomografía computarizada de haz cónico ha permitido la valoración tridimensional del defecto, consideraciones sobre la dosis de radiación para el paciente y artefactos de imagen que puedan limitar su uso extensivo en esta tarea diagnóstica han conducido a la investigación sobre la utilidad de algoritmos de reducción de artefactos y protocolos de adquisición de imagen de baja dosis de radiación.

## Fuente de financiamiento:

Autofinanciado
